# Open conversion in laparoscopic cholecystectomy and bile duct exploration: subspecialisation safely reduces the conversion rates

**DOI:** 10.1007/s00464-021-08316-1

**Published:** 2021-02-02

**Authors:** Ahmad H. M. Nassar, Hisham El Zanati, Hwei J. Ng, Khurram S. Khan, Colin Wood

**Affiliations:** 1 Department of Surgery, University Hospital Monklands, Airdrie, Lanarkshire, UK; 2 Department of Surgery, University Hospital Hairmyres, East Kilbride, Lanarkshire, UK; 3 Department of Surgery, NHS Greater Glasgow and Clyde, Glasgow, UK; 4 Laparoscopic Biliary Service, University Hospital Monklands, Monkscourt Avenue, Airdrie, Lanarkshire, Scotland ML6 0JS UK

**Keywords:** Laparoscopic cholecystectomy, Conversion, Difficult cholecystectomy, Nassar scale, Fundus first cholecystectomy, Subtotal cholecystectomy

## Abstract

**Background:**

Open conversion rates during laparoscopic cholecystectomy vary depending on many factors. Surgeon experience and operative difficulty influence the decision to convert on the grounds of patient safety but occasionally due to technical factors. We aim to evaluate the difficulties leading to conversion, the strategies used to minimise this event and how subspecialisation influenced conversion rates over time.

**Methods:**

Prospectively collected data from 5738 laparoscopic cholecystectomies performed by a single surgeon over 28 years was analysed. Routine intraoperative cholangiography and common bile duct exploration when indicated are utilised. Patients undergoing conversion, fundus first dissection or subtotal cholecystectomy were identified and the causes and outcomes compared to those in the literature.

**Results:**

28 patients underwent conversion to open cholecystectomy (0.49%). Morbidity was relatively high (33%). 16 of the 28 patients (57%) had undergone bile duct exploration. The most common causes of conversion in our series were dense adhesions (9/28, 32%) and impacted bile duct stones (7/28, 25%). 173 patients underwent fundus first cholecystectomy (FFC) (3%) and 6 subtotal cholecystectomy (0.1%). Morbidity was 17.3% for the FFC and no complications were encountered in the subtotal cholecystectomy patients. These salvage techniques have reduced our conversion rate from a potential 3.5% to 0.49%.

**Conclusion:**

Although open conversion should not be seen as a failure, it carries a high morbidity and should only be performed when other strategies have failed. Subspecialisation and a high emergency case volume together with FFC and subtotal cholecystectomy as salvage strategies can reduce conversion and its morbidity in difficult cholecystectomies.

After the first laparoscopic cholecystectomy was performed by Mühe in Germany in 1986, the procedure became one of the most common surgical procedures performed worldwide [1].

Conversion from laparoscopic cholecystectomy (LC) to open cholecystectomy (OC) may be resorted to for various reasons with reported rates of 1% to 15% [2, 3]. Open conversion increases the operative time, complication rates, perioperative costs and the length of hospital stay [4–6].

Difficult cholecystectomies are usually associated with severe inflammation that distorts the anatomy and renders dissection more difficult (i.e. acute cholecystitis, empyema, gangrene, perforation and Mirizzi syndrome) or with liver cirrhosis increasing the risk of bleeding and a higher probability of conversion. As laparoscopic skills increase surgeons become more able to utilise different techniques to reduce their conversion rates. Some strategies were already well established in OC such as fundus first dissection (FFD) and subtotal cholecystectomy [7].

Acute cholecystitis was once considered a contraindication to LC [8]. However, the current guidelines of several international societies suggest that these patients should be offered an early LC [9]. Surgeons are thus likely to be exposed to more difficult gall bladders with the potential for an increase in open conversion.

## Methods

Prospectively collected data from 5738 LC performed by a single surgeon (AHMN) or his trainees under direct on-table supervision over 28 years were reviewed. Data on patient demographics, type of admission, clinical presentation, radiological findings, interval from admission to surgery, operative difficulty grade, operative time, perioperative complications, re-admissions and mortality were recorded.

Cases that underwent conversion to open cholecystectomy were identified and their preoperative, operative and postoperative data were analysed.

This biliary firm managed, by protocol, most referrals of biliary emergencies within the hospital and occasionally inter-hospital transfers. A minimum emergency workload of 60% is agreed according to the consultant’s job plan. The unit adopts a policy of intention to treat during the index emergency admission and single session laparoscopic management of bile duct stones.

We do not routinely rely on preoperative MRCP or ERCP to investigate or treat patients with suspected bile duct stones. However, cross-sectional imaging is carried out when any patients presenting with jaundice have any risk factors to suspect malignancy. We perform routine intraoperative cholangiography (IOC) and, when indicated, laparoscopic bile duct exploration (LCBDE). IOC helps clarify the anatomy of the biliary tree particularly in difficult cases. We use the Nassar difficulty grading scale to document operative difficulty of the cholecystectomy. This has been shown to standardise the description of operative findings by different surgeons in order to facilitate audit, training assessment and research. It provides a tool for reporting disease severity and technical difficulty and can be utilised to reliably compare outcomes according to case mix and operative complexity [10].

### Operative technique and strategies for difficult LC

**Fig. 1:**
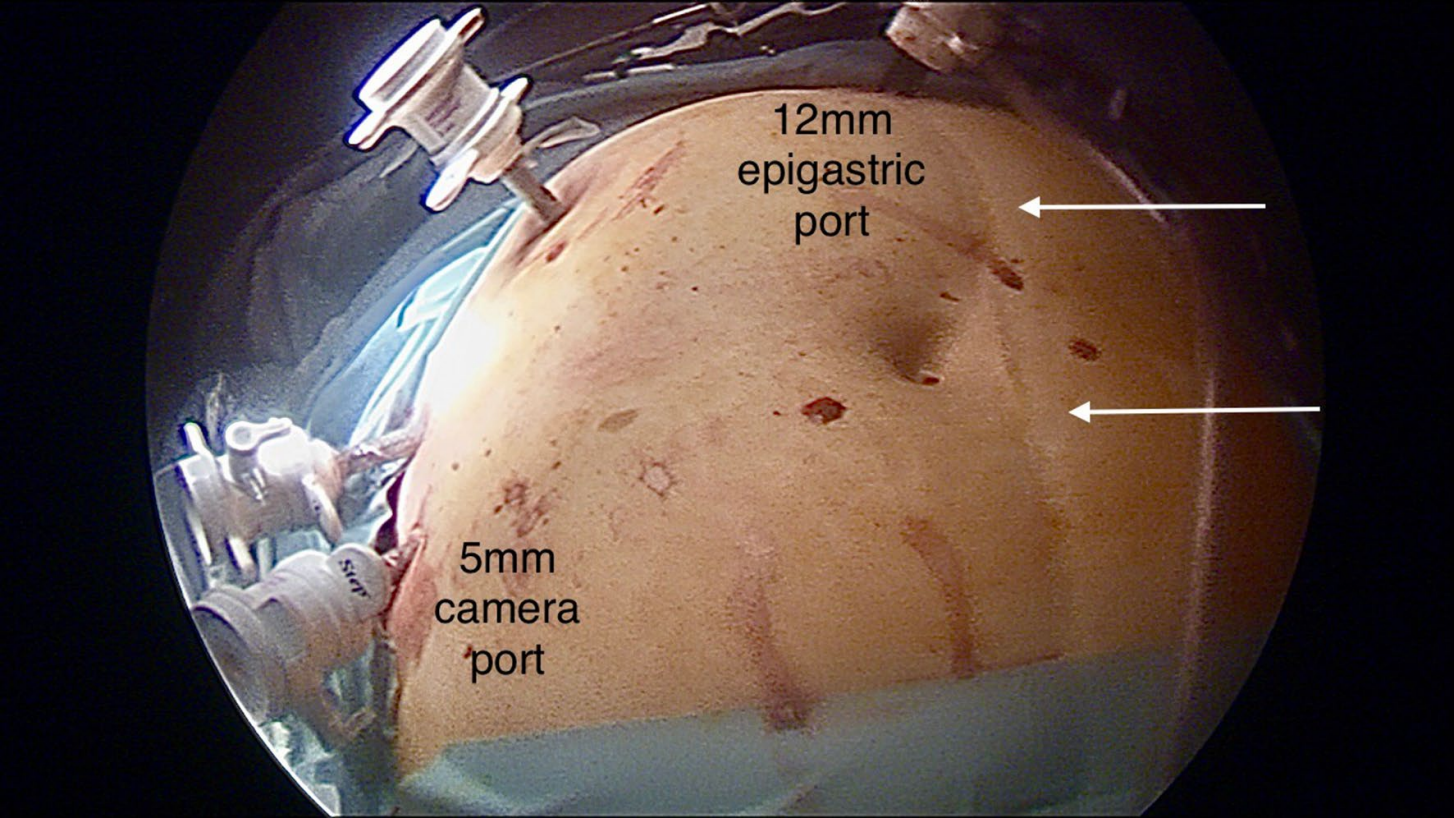
Modified epigastric access avoiding a midline scar and adhesions in the centre of the abdomen using a lateral camera port

A four port technique is employed in the American position. Modified open access is established through an infraumbilical 11–12 mm port and three 5 mm epigastric, sub-costal and right flank ports. The first access port should be inserted at a different point to avoid abdominal scars, usually at the epigastrium. Adhesions between the gallbladder and omentum or bowel loops were divided using sharp or blunt dissection. Bowel adhesions are divided at the interface with parietal peritoneum, or with the gallbladder or liver. Adhesiolysis is limited to the minimum needed to clear the port sites and sweep bowels away from the operating field and subsequently camera ports do not need to follow the classical distribution (Fig. 1). The cystic pedicle is dissected using a blunt “duckbill dissector” (Karl Storz, Tutlingen, Germany). The diathermy hook was abandoned after the first few cases and has no place in our practice. As most conversions occurred in the early part of the series, dissection of the Calot’s triangle employed the infundibular view approach. A policy of routinely seeking to display the critical view of safety (CVS) was adopted in the last 5 years.

Once the gallbladder/cystic duct junction and the cystic artery were identified and isolated and the CVS obtained, the neck of the gallbladder was ligated using 2–0 absorbable suture. The cystic duct was incised close to the gallbladder neck, a cholangiography catheter (Cook Medical INC, USA) was inserted into a cholangiography cannula (Karl Storz, Tutlingen, Germany) through the right sub-costal port and operative cholangiography was obtained [11]. Once the anatomy of the main bile ducts was clarified and CBD stones were excluded, the cystic duct was ligated using absorbable 2–0 suture material and divided. The use of metal clips to secure the cystic duct was abandoned 23 years ago. Gallbladder separation was then carried out using the “duckbill dissector”, opening windows in the peritoneal reflection, the jaws creating a sub-serosal plane and sweeping the gallbladder away from the liver.

**Fig. 2:**
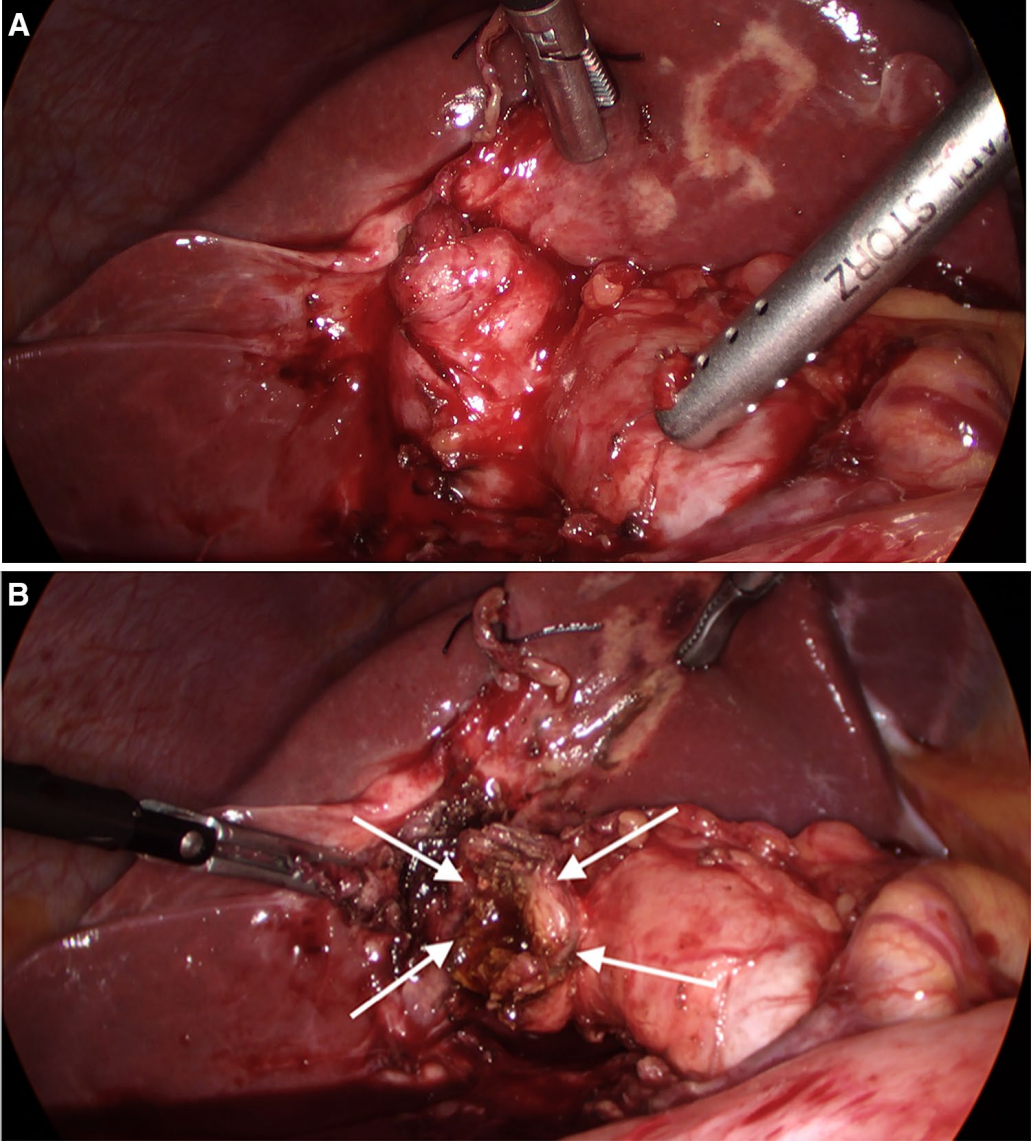
**A** A contracted gallbladder with a sessile junction with the common bile duct may make it impossible to display the critical view of safety. **B** FFD culminating in the "funnel method' facilitating safer and further posterior dissection to achieve a complete cholecystectomy

When a difficult cholecystectomy is encountered, the difficulty grade is determined soon after trial dissection and a first time-out is used to decide on which techniques are used to facilitate further dissection, aiming at safely avoiding conversion. A tense or thick-walled gallbladder (acute cholecystitis (AC), empyema, mucocele) is decompressed. Grasping the gallbladder may occasionally be facilitated by making a small incision at the fundus to insert one of the grasper’s jaws. A packed gallbladder may be opened and evacuated. Hartmann’s Pouch stones (HPS) were either pushed back into the gallbladder or occasionally removed to facilitate the dissection of the cystic pedicle or cystic plate. An effort is made to identify the cystic lymph node as a marker of the underlying cystic artery. A sub-serosal approach at the Hartman’s pouch or the body of the gallbladder facilitates dissection close to the wall of an inflamed thick-walled gallbladder [12]. When a contracted gallbladder is encountered, dissection around the body of the gallbladder or FFD is considered. FFD stops short of the area of the right hepatic artery/duct and transvesical IOC through the body or infundibulum of the gallbladder is performed, especially if a Mirizzi Syndrome abnormality was suspected [13]. If IOC could not be obtained, the gallbladder body is divided horizontally creating a “funnel-shaped remnant” presenting the whole contour of the Hartman’s pouch and allowing safe blunt posterior dissection (Fig. 2A and B). In our practice, this helps to achieve a complete laparoscopic cholecystectomy, reducing the incidence of subtotal cholecystectomy (SC) which was rarely resorted to in this series.

Informed consent was obtained from all patients with specific emphasis on the specialisation of the unit with regard to the management of suspected bile duct stones. IRB approval was not required as the management protocols were in line with the recommendations of national and international societies. Statistical analysis: Qualitative data were given as frequency and percentages. The *p* values and odds ratio with 95% confidence interval for categorical values were calculated using two-tailed Fishe’s exact test. *p* values < 0.05 were considered statistically significant.

## Results

**Fig. 3:**
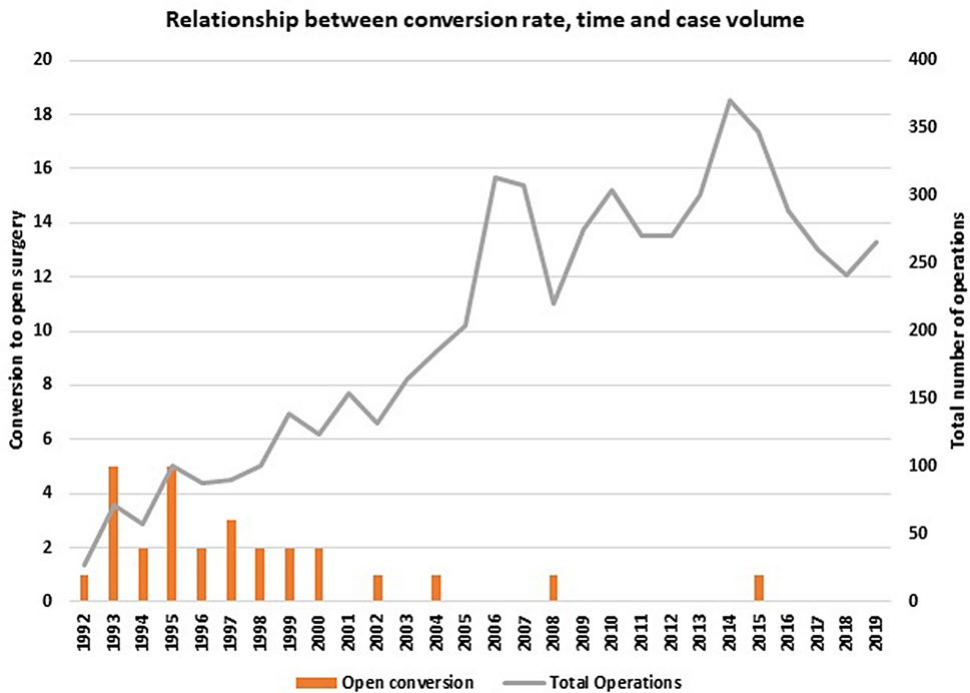
Relationship between conversion rate, time and case volume

28/5738 patients underwent conversion to open cholecystectomy (0.49%). Most of the conversions occurred in the earlier part of our series as shown in Fig. 3. This shows the effect of the learning curve and the case load on the development of experience, subspecialisation and increased skill in dealing with complex cases. Half the conversions in this series occurred in the first four years (8/261 cholecystectomies and 6/24 bile duct explorations). 10 of the converted patients (37%) were males (a conversion rate for male patients in the series of 0.7%) and 18 were females (conversion rate of 0.4%). Age ranged from 26 to 78 years with a mean age of 55 years. The conversion rate for male patients 60 years of age or over was 0.76%.

9 of the procedures (32%) were elective admissions and 19 were emergencies (68%). The median admission to surgery interval in the emergency admissions was 3 days (range 1–10 days). 7 patients were referred following investigations by other surgeons or from other hospitals, resulting in 3 of the longest referral to surgery intervals.

5 patients had previous abdominal surgery (17.9%) and 6 had documented previous episodes of cholecystitis (21.4%). The conversion rates relative to specific traditional risk factors are shown in Table 1. The only significant predictors of conversion in this cohort were emergency admission, presenting with jaundice, a history of previous acute cholecystitis and a dilated CBD on ultrasound scan. 14 patients in this cohort presented with jaundice. Only one had undergone an ERCP and CT reported as showing CBD stones but was found at surgery to have an empyema of the gallbladder and a cholangiocarcinoma requiring a hepaticojejunostomy. None of these 14 jaundiced patients had an MRCP as 12 were treated in the early part of the series before MRCP became available and the last two were young patients with no risk factors for cancer.

**Table 1:** Conversion rates relative to specific preoperative risk factors

Preoperative risk factors	Risk factor positive no	Conversion no (%)	Risk factor negative no	Conversion nso (%)	*p* value	OR (95% CI)
Age ≥ 60 years	1859	13 (0.7%)	3879	15 (0.38%)	0.154	1.814 (0.861, 3.20)
Male aged ≥ 60 years	656	5 (0.76)	830	5 (0.6%)	0.757	1.267 (0.365, 4.396)
Emergency admission	2551	19 (0.74%)	3187	9 (0.28%)	0.020	2.650 (1.197, 5.867)
Acute cholecystitis	506	3 (0.6%)	5232	25 (0.47%)	0.733	1.242 (0.374, 4.129)
Jaundice	1043	14 (1.3%)	4695	14 (0.29%)	< 0.001	4.559 (2.162, 9.571)
Previous cholecystitis	328	6 (1.8%)	5410	22 (0.4%)	0.004	4.577 (1.843, 11.367)
Previous jaundice	304	2 (0.65%)	5434	26 (0.48%)	0.659	1.377 (0.325, 5.831)
USS thick or contracted GB	872	7 (0.8%)	4866	21 (0.43%)	0.180	1.867 (0.791, 4.405)
USS Dilated CBD	937	12 (1.28%)	4801	16 (0.33%)	0.001	3.880 (1.829, 8.228)
Previous abdominal surgery	1759	5 (0.28%)	3979	23 (0.57%)	0.156	0.490 (0.186, 1.292)
Risk factors for CBD stones	2047	19 (0.93%)	3691	9 (0.24%)	0.001	3.833 (1.731, 8.487)

Of the 680 patients found at operation to have acute cholecystitis or empyema of the gallbladder, 6 were converted (0.8%). However, two of these conversions were due to a suspicion of malignancy and one was due to an impacted stone in a Mirizzi Type 1 case. Only three were caused by the condition of the gall bladder, a conversion rate similar to the rest of the series. In the whole series, 16 patients who underwent laparoscopic bile duct explorations had undergone previous biliary interventions: 9 open cholecystectomies and 7 laparoscopic cholecystectomies. 4 patients who had previous cholecystectomies were subsequently optimised and had laparoscopic cholecystectomies (patients who remained unfit for surgery are not part of the study). None of the patients with previous biliary procedures required open conversion.

16 conversions (57%) occurred in patients who underwent 1318 CBD exploration, a 1.2% conversion rate. The most common causes of conversion in our series were dense adhesions in 9 patients (32%), impacted CBD stones in 7 (25%), 3 of which could not be removed at open surgery and required a choledochoduodenostomy and Mirizzi syndrome in 4 cases.

The causes of conversion in this series are shown in Table 2. At operation, adhesions between the gallbladder and the hepatic flexure were reported in 18 patients and between the gallbladder and the duodenum in 11 and to both viscera in 9. The cystic pedicle area was reported as difficult in 23 cases with 20 patients (71.5%) being difficulty grade 4 or 5. 6 of the 173 patients subject to FFD had open cholecystectomy. However, all of the last 135 FF cholecystectomies performed over the last 20 years were completed laparoscopically.

**Table 2:** Causes of open conversion

Main reason for conversion	Number of patients
Impacted CBD stones (Non Mirizzi)	7 (25%)
Adhesions GB to omentum, hepatic flexure, duodenum	7 (25%)
Adhesions. Distant. Bowel injury	2 (7%)
Mirizzi ( includes 1 with impacted CBD stone)	4 (14%)
Failure to establish pneumoperitoneum	2 (7%)
Bleeding, liver cirrhosis	1 (3.5%)
Suspicion of malignancy	1 (3.5%)
Cholecystoduodenal fistula	1 (3.5%)
Unclear anatomy	1 (3.5%)
Slipped T tube after LCBDE	1 (3.5%)
CBD stricture (cholangiocarcinoma)	1 (3.5%)

The median duration of surgery was 195 min (70–420). The median duration for the whole series was 71 min for LC and 119 min in the 1318 patients who underwent CBD exploration.

The total hospital stay ranged from 4 to 95 days with a median of 11 days. 16 converted patients had bile duct exploration earlier in the series. Their longer hospital stay (median 14 days, range 7–30) reflected the need for biliary drains in 11 patients, bilioenteric anastomosis in 6 and 6 having had postoperative complications including retained stones requiring ERCP in 3 patients. The median number of admission episodes was 1; only 6 patients had two admissions and 3 had three, including all previous admissions and re-admissions. The presentation to resolution period ranged from 1 to 30 weeks with a median of 2.

Morbidity within this group of patients was relatively high with a complication rate of 32%. The different complications encountered, their management and outcome are summarised in Table 3. There were no deaths.

**Table 3:** Complications encountered in the conversion patients in our series and their management

Complication	Number	Readmission	Re-intervention	Clavien–Dindo classification	Hospital stay/days
Retained stones	3 (10.7%)	1	3 ERCP	G IIIa	24, 14, 10,
Abdominal collections	2 (7%)	1	1 P/C Drain 1 conservative	G IIIa G II	30 21
Bile leak	1 (3.5%)		Conservative	G I	12
Intestinal fistula/abdominal collection	1 (3.5%)	1	P/C drain, settled	G IIIa	95
Chest infection	1 (3.5%)		Conservative	G II	7
Bile leak due to cholangiocarcinoma	1 (3.5%)		ERCP	G IIIa	21

During the period of this study, no patients were preselected for open cholecystectomy on account of expected difficulty, e.g. previous abdominal surgery or confirmed bile duct stones. Three patients had cholecystectomies during two open gastrectomies and one splenectomy. *However, four patients were referred to a liver surgery unit for biliary reconstruction immediately after LC; two with bile duct injuries and two with Mirizzi Type III and IV*. All underwent open surgery within 24 h of the original laparoscopic procedures.

## Discussion

The incidence of open conversion in the literature is subject to wide variations in the clinical presentations, experience of the surgeons and the gallbladder pathology they encountered. The conversion rate in our series was just under 0.5% in spite of a 44% incidence of emergency admissions and a high rate (23%) of laparoscopic CBD explorations (LCBDE). Subgroup analysis of 12 conversions in our 4426 patients undergoing LC without LCBDE, reflecting the current practice of staged management of bile duct stones, would result in an adjusted conversion rate of 0.27%.

The CholeS study was a large prospective study assessing outcomes and variations in practice of cholecystectomy performed across the United Kingdom in 8820 patients in March and April 2014 [14]. The conversion rate of 3.4% may therefore reflect current practice and the different causes of conversion in the United Kingdom.

### Predictors of conversion

Various preoperative and operative risk factors for open conversions have been suggested. The CLOC score (**C**onversion from **L**aparoscopic to **O**pen **C**holecystectomy) derived from the CholeS study database identified older age, American Society of Anaesthesiologists (ASA) classification, male gender, indication for surgery, CBD diameter and gallbladder wall thickness as significant independent predictors of conversion on multivariate analysis [15]. The type of admission and preoperative ERCP were predictive of conversion on univariate analysis but not on multivariate analysis. Although our 152 patients with preoperative ERCP had higher grades of operative difficulty, none were in the conversion group. The Body Mass Index (BMI) was not a risk factor for conversion in either study.

The indication for surgery scoring highest in the CLOC score was the presence of CBD stones. This mirrors the results of our series. The conversion rate for those presenting with jaundice was 1.3%, compared to 0.29% for non-jaundiced patients: *p* < 0.001. However, while 57% of our converted patients underwent CBD explorations, there were only 12% in the CholeS study. It was not clear whether, at least in some cases, this was the result of the need to convert to deal with unexpected bile duct stones or of difficulties encountered during planned laparoscopic CBD explorations necessitating conversion. CholeS reported a total of 557 patients with bile duct stones (6.3%) versus 1318 (23%) in this series. The preoperative characteristics predictive of conversion in this study vs. the national study are summarised in Table 4.

**Table 4:** Preoperative characteristics predictive of conversion in our series vs. CholeS study

	This study	CholeS	*p* value	OR (95% CI)
Conversions	28/5738 (0.49%)	221/6615 (3.34%)*	0.00001	0.142 (0.096–0.211)
Age ≥ 50 years	17 (60.7%)	179 (81%)	0.025	0.363 (0.158–0.831)
Male gender	10 (35.7%)	111 (50%)	0.165	0.551 (0.243–1.246)
Emergency admission	18 (64.3%)	57 (26%)	0.0001	5.179 (2.259–11.873)
Preoperative ERCP	0%	57 (26%)	0.001	Not applicable
Main indication for surgery				
Acute Cholecystitis	3 (10.7%)	123 (56%)	0.001	0.096 (0.028–0.326)
Pancreatitis	0%	13 (6%)	0.3714	Not applicable
CBD stone/jaundice	13 (46.4%)	40 (18%)	0.002	3.922 (1.731–8.885)
Thick-walled gall bladder on ultrasound	7 (25%)	122 (55%)	0.0043	0.27 (0.110–0.662)
Dilated CBD on ultrasound	11 (39.3%)	66 (30%)	0.385	1.520 (0.675–3.420)

*CholeS data were divided into two random groups with similar conversion rates: one to produce a risk score and one to validate the resulting score

Overall morbidity of the converted patients in the CholeS study (33%) was similar to ours. However, while the operative difficulty grading was comparable, our median operative time and hospital stay were longer than in the CholeS study for the converted patients (Table 5). This is likely due to the high incidence of bile duct exploration in our study as our “LC only” conversions have a median operative time of 130 min and a hospital stay of 8 days, both similar to CholeS.

**Table 5:** Operative parameters and postoperative outcomes of conversions

	This study no = 5738	CholeS no = 8820	*p* value	OR (95% CI)
No of conversions	28 (0.49%)	297(3.37%)	0.00001	0.146 (0.099–0.215)
Nassar difficulty grade				
IV + V	20 (71.4%)	212 (71.4%)	1	1.002 (0.425–2.363)
III	4 (14.2%)	65 (21.8%)	0.4708	0.595 (0.199–1.776)
II	2 (7.1%)	12 (4%)	1	1.827 (0.388–8.606
I	2 (7.1%)	7 (2.3%)	0.1674	3.187 (0.629–16.134
Median operative time	195 min	120 min		
Median Hospital stay	11 days	6 days		
Mortality	0%	2 (0.7%)	1	Not applicable
Total morbidity	9 (32%)	98 (33%)	0.8321	0.962 (0.420–2.204)
Bile leak	1 (3.5%)	25 (8%)	0.7096	0.403 (0.053–3.091)
Bile duct injury^a^	0%	6 (2%)	1	Not applicable

^a^Two bile duct injuries occurred in this series but were not converted. They underwent open biliary reconstruction at a liver surgery unit within 24 h. Satisfactory symptom-free follow-up of 8 and 15 years

Shamieh et al. [16] reported a conversion rate of 5.4% in 5048 patients, with acute cholecystitis accounting for 29% of conversions followed by unclear Calot’s triangle anatomy in 17% and dense adhesions in 14%. CBD exploration accounted for only 3.7% of conversions in that institution where laparoscopic ductal exploration was not available. In addition, 11% were preselected for open cholecystectomy without a laparoscopic attempt.

Tuveri et al. [17] reported a 3.6% conversion in 1965 patients. Acute cholecystitis of a duration > 72 h, BMI of over 30 and patients with previous gastric surgery were excluded. FFD was the main focus of the study with 29 attempted and 6 converted. 6 patients had CBD stones, 4 dealt with laparoscopically and 2 converted. It was not clear how CBD stones in the non-FFD group were dealt with. The reasons for conversion were Mirizzi syndrome, Cholecystoduodenal fistula, dense adhesions and short, wide cystic duct.

### Conversion in laparoscopic bile duct exploration

A meta-analysis of 13 studies including 872 LCBDE [18] reported a conversion rate of 4.1%. No details of the exact causes of conversion were given. Another meta-analysis [19] comparing transcystic and transductal CBD exploration in 26 studies including 3396 patients reported conversion rates of 3.2% and 2.4% for these exploration modalities, respectively, with no details of the causes of conversion. Our overall conversion rate was 1.2%: 3/871 (0.34%) for transcystic and 13/447 (2.9%) for transductal exploration.

Paganini et al. [20] reported 344 LCBDE with a conversion rate of 4.4%, the cause of conversion being dense adhesions in 33% and impacted stones in 20%. Our series of 1318 LCBDE with 16 conversions had impacted CBD stones in 6 (37%), adhesions in 4 (25%), need for hepaticojejunostomy in 3 (19%) and miscellaneous causes in 3. Once beyond the learning curve, there were only four conversions in the last 1243 ductal explorations, a conversion rate of 0.32% not dissimilar to that for LC alone. The learning curve for bile duct exploration was also reported by Paganini et al. as the reason for 3 of their 15 conversions.

Conversion rates improve with growing experience and case volume (Fig. 3). Most conversions occurred in the first 7 years. Specialising in biliary emergencies, routine cholangiography, refining the techniques for difficult laparoscopic bile duct explorations and a trend towards using FFD in difficult LC reduced conversions to 8 of the last 5198 cases (0.15%) over 22 years.

### Salvage strategies

173 patients underwent laparoscopic FFD (6 converted) and 6 had laparoscopic SC, 85% graded IV–V on the Nassar difficulty scale. FFD was performed when the cystic duct pedicle was encased in dense adhesions, when the cystic artery and duct could not be separated, when Hartmann's pouch was found to be densely adherent to the common bile duct, and when the presence of a Mirizzi abnormality was suspected. We have previously described the technique of FFD and its effect on the conversion rate [21].

Subtotal cholecystectomy and FFD were proposed as important strategies in dealing with difficult LC in several studies reporting different outcomes [22–25]. The fenestrating SC technique is preferred by most authors [26]. However, the, risks of bile leakage and of stones left in the gallbladder remnant are a cause for concern, whether the fenestrating or the reconstituting method is used. In this series, we rarely resorted to subtotal cholecystectomy, excising as much of the gallbladder as possible, ensuring the removal of all stones from the cystic duct stump and performing cholangiography or choledochoscopy to avoid retained CBD stones. Table 6 compares the outcomes of our series and some studies with similar characteristics in relation to the effect of FFD and SC on the conversion rates. However, unlike in our series, some authors have preselected patients for open cholecystectomy excluding cases from LC on the grounds of expected difficulty or due to the presence of bile duct stones.

**Table 6:** Comparison of the outcomes of fundus first dissection & subtotal cholecystectomy

	No of LC cases in series	Incidence of FFD/SC	Total conversion rate	Potential conversion without FF/SC	IOC with FFD/SC	Overall complication rate with FFD/SC (%)	Residual CBD stones after FFD/SC (%)	Lap CBD exploration
Nassar et al. This study	5738	2.8%/0.08%	0.49% (+ 3 open from start—0.052%)	3.5%	93.8%	12.6	1.2	Yes
Hubert et al. [23]	500 (elective only)	7.1%	2% (+ 52 open from star—9%)	25.6%	79.5%	15.4	5.1	No
ElShaer et al. [25]	1231	100% subtotal (meta-analysis)	8% (+ 19% open from start)	8%	4/30 studies	27	3	No
Sormaz et al. [7]	213	6.2%/2.8%	1.4% (+ 27 open from start excluded—11%)	0% but 46% of FFD had SC	no	15	7.5	No
Gupta et al. [24]^a^ (FFD only not SC)	145	18.6% in difficult LC	2% (excluded fistulas, CBD stones, cancer)	4.1%	no	1.5	0	No
Tuveri et al. [17]	1965 (> 72 h AC* excluded)	1.5%	3.4%	20%	60%	20	6.9	Yes

^a^Gupta randomised 31% LC to FFD preoperatively regardless of difficulty and resorted to FFD due to difficulty in an additional 18.6% in the conventional LC group

Although CBD stones caused most conversions in this series, only two conversions were needed in the last 1150 to perform biliary enteric anastomosis for a Mirizzi Type 2 and an impacted stone at the ampulla. Stone fragmentation using biopsy forceps, ultrasound lithotripsy and laser lithotripsy facilitated laparoscopic completion of 118 cases with impacted CBD stones. The utilisation of choledoscopy is essential in all cases.

Mirizzi syndrome caused 4 conversions in this cohort, 3 Type I and 1 Type II [13]. CBD stones encountered in a Type III and a Type IV were dealt with laparoscopically but the patients were subsequently referred to liver surgery units for biliary reconstruction. Subspecialisation helped to achieve definitive one-session laparoscopic treatment in 89% of the 58 Mirizzi Syndrome patients in our series.

## Conclusion

Open conversion should not be regarded as a complication or a failure in laparoscopic cholecystectomy. It is occasionally the safest option for the patient when encountering a difficult gallbladder. Nevertheless, conversion still carries a high complication rate and it is good practice to consult a more experienced surgeon where available before resorting to conversion. FFD and SC are useful salvage techniques which are proven to reduce the conversion rate. Subspecialisation in managing biliary emergencies, with or without single stage management of bile duct stones, can reduce the conversion rates in difficult cholecystectomies and bile duct explorations.
